# No statistically significant difference in long term scarring outcomes of pediatric burns patients treated surgically vs. those treated conservatively

**DOI:** 10.3389/fsurg.2022.727983

**Published:** 2022-09-09

**Authors:** Riyam Mistry, Fadi Issa

**Affiliations:** Nuffield Department of Surgical Sciences, University of Oxford, Oxford, United Kingdom

**Keywords:** scarring, paediatric, surgery, conservative treatment, long term outcome

## Abstract

**Introduction:**

Paediatric burns are a common clinical presentation. The long-term scar outcomes in paediatric burns patients are relatively unknown as most are discharged after 6 weeks follow up, apart from the small number that are followed up by scar services depending on geographical availability. We aimed to determine whether the long-term scarring outcomes are significantly different in those who had surgical treatment with Versajet® debridement and Biobrane^®^, vs. those treated conservatively with non-adherent Mepitel^®^ and Acticoat^®^ dressings, in a cohort of paediatric burns patients.

**Methods:**

The parents of all paediatric burns patients admitted to Stoke Mandeville Hospital from October 2014 to September 2017 were contacted by telephone to fill in the paediatric Brisbane Burn Scar Impact Profile (BBSIP), the only patient reported outcome measure (PROM) specifically aimed at children. The results from the questionnaires underwent statistical analysis to see if there was a significant difference in questionnaire scores between children treated surgically vs. those treated conservatively.

**Results:**

A total of 107 children were admitted in the timeframe, responses were received from 34 patients with 13 having been treated surgically and 21 having been treated conservatively. In all 58 questions that make up the BBSIP, there was no statistically significant difference observed in the scores of those treated surgically vs. those treated conservatively. For 31 questions on the BBSIP, the lowest score indicating the best outcome was observed in all patients in both groups.

**Discussion:**

Surgical management for burns is always the last resort. Our results could be interpreted to suggest clinicians need not fear the longer-term impact a scar may have when deciding whether to treat a paediatric burns patient surgically or conservatively. This study is the first to assess longer-term scar outcomes using the BBSIP. A larger data set and comparison with other burn units in the UK may help to provide more information on scar outcomes between different methods of surgical and conservative treatment. We found no statistically significant difference in the long-term scar outcomes as assessed by the BBSIP in paediatric burn patients treated with Versajet^®^ debridement and Biobrane^®^, vs. those treated conservatively with non-adherent Mepitel^®^ and Acticoat^®^ dressings.

## Introduction

The global number of pediatric burns presenting to hospitals in 2005 was 505,276, with a then pediatric population of 1.843 billion (1). There is no defined “gold standard” for treating pediatric partial thickness burns. First aid involves cooling the site with water to dissipate any thermal energy followed by providing a moist wound environment, physiologically stabilizing the patient, removing exudate, preventing infection, and minimizing pain (2, 3).

The Stoke Mandeville Hospital Burns unit receives approximately 450 pediatric burns referrals per year. The most common presentation is of scald injuries to the chest, typically from hot drinks. The thinner dermis in younger children can often result in a deeper more substantial burn compared to a similar injury in adults (2). The majority of patients can be treated conservatively, although those with deeper burns may require surgical intervention.

Depending on the depth of the burn, a surgical approach may be required to remove necrotic non-viable tissue. An issue with this surgical debridement is removal of normal healthy viable tissue along with non-viable tissue which can result in worse scarring (4, 5). The Versajet^®^ (Smith and Nephew, Key Largo FL, USA) hydrosurgery system has been used as a burn debridement method for 12 years (6). Its mechanism is to use high pressure sterile saline to create a controlled cutting field with a built in suction system. Due to its ability to debride in a controlled manner, it is regarded as a useful tool for debriding burned tissue whist preserving the dermis (7). The thinner dermis observed in the pediatric population means this greater level of control is advantageous (5). Cubison et al 2006 reported using Versajet^®^ debridement and subsequent Biobrane^®^ dressings in pediatric patients with good healing and scar outcomes (5).

After the burn has healed, injuries that have gone beyond 33.1% of skin depth will typically leave a scar (8). Scars can be itchy, painful, tight, functionally restrictive, and cosmetically disfiguring. Many studies have reported on the short term outcomes of pediatric burn wounds focusing on factors such as hospital stay, time to healing and cost-effectiveness of treatment (2, 3, 9–11). Longer term scar outcomes are less commonly described, with discharge from outpatient clinic reported as a common contributory factor (2). Fan et al 2018 commented on the longer term scarring outcomes of pediatric burns treated with Biobrane^®^ compared to those treated with silver based dressings (9). No difference in outcomes was observed when using the patient reported outcome section of the Patient Observer Scar Assessment Scale (POSAS) (9). The POSAS is a scar specific assessment tool comprised of an observer section, typically filled in by a clinician; and a patient reported section for scar symptoms and appearance. For Versajet^®^ debridement, there is limited literature observing the longer term scarring outcomes in pediatric burn patients. Legemate et al 2018 put forward a research protocol to compare the longer-term scarring outcomes in patients of all ages with burns treated with conventional surgical debridement vs. Versajet^®^ debridement (12). Although results have not been published yet, they will be following up patients at 12 months post-surgery using the POSAS as the primary outcome measure.

The novel BBSIP is a PROM developed to assess scar outcomes based on elements related to quality of life in patient with scars from burn injuries (13). The BBSIP has been further developed to create versions dedicated to young children aged 8 to 18 (BBSIP_8–18_) and the caregivers of younger children aged 0 to 8 (BBSIP_0–8_) (14, 15). A recent study measured the internal consistency, test-retest reliability, longitudinal validity and responsiveness of the BBSIP in post-acute burn period (15). The content and construct validity had been reported in another study (13, 16). The authors concluded that the BBSIP is a suitable PROM in the post-acute burn period and that future studies will help evaluate its use as a longer term scar outcome tool (15). The POSAS is also considered to be a well validated PROM for scarring. The observer element filled in by an assessor has demonstrated acceptable reliability for the vascularity section and total score; along with internal consistency and construct validation (17, 18). Test-retest reliability of the POSAS has been reported as acceptable for all items (except vascularity and relief) in the observer section and the pain section of the patient version (19). The internal consistency for the patient scale has been reported as generally acceptable (17).

There is little data on the outcomes of the scar in children treated conservatively vs. surgically for burns. By utilizing a purpose created paediatric burn scar assessment tool; the aim of this study was to determine if there were any long-term differences in scarring outcomes of pediatric burn patients treated with surgical Versajet^®^ and Biobrane^®^ compared to conservative Acticoat^®^ and non-adherent dressings. By comparing the two, we hoped to determine if there would be superior scarring outcomes in either group.

## Materials and methods

A retrospective review was performed of all pediatric burns patients admitted to the Stoke Mandeville Burns Unit from October 2014 to September 2017. At the time of this study, the unit's policy for superficial partial thickness burn conservative burn treatment was non-adherent dressing Mepitel^®^ and Acticoat^®^ after cleansing with aqueous chlorhexidine. Surgical management for superficial partial thickness burns was with Versajet^®^ debridement and Biobrane^®^ dressings.

Established for over 30 years, Biobrane^®^ (UDL Laboratories, Rockford, IL, USA) is a popular treatment choice for superficial and partial thickness burns. It consists of a semipermeable silicone membrane combined with a Type I collagen (of porcine origin) coated nylon mesh. Multiple studies have demonstrated that Biobrane^®^ can protect against infection, reduce pain, improve healing time resulting in a shorter inpatient stay, reduced dressing changes and better cost-effectiveness (10, 20–25) These qualities have made it a popular dressing choice around the UK. Despite its widespread use, there is a lack of data on longer term scar outcomes of patients treated with Biobrane (10).

Silver sulfadiazine and silver nitrate impregnated dressings such as Acticoat^®^, are another popular treatment for partial thickness burns (22, 23, 26). The silver is reported to provide an anti-microbial surface helping to reduce the risk of infection of the burn wound (27). Acticoat^®^ is a popular choice in UK burn centers, consisting of a silver nano-crystalline mesh that can be manipulated over the burn site (27, 28). Other reported benefits of Acticoat^®^ include fewer inflammatory reactions compared to silver sulfadiazine and prolonged silver release (27, 28). There is conflicting information between topical silver dressings compared with Biobrane^®^, but the few available studies report no observed differences in longer term outcomes (9, 11, 26).

A total of 107 patients satisfied our inclusion and exclusion criteria (Table 1) and were included in the study. The data collected from the patients' online medical records included: age, gender, Total Body Surface Area (TBSA), mechanism of burn (e.g., scald), number of follow up appointments, further treatments, the location of the burn, injury date, assessment date, admission date and treatment received. As the focus was on longer term scar outcomes, many patients were no longer being followed up in outpatient clinic and so scar evaluation was conducted *via* telephone interview using the age-appropriate version of the BBSIP. The 107 patients were contacted a maximum of 3 times using telephone numbers from their records.

**Table 1:** Inclusion and exclusion criteria.

Inclusion Criteria	Exclusion Criteria
– All paediatric burn referrals requiring admission – Patients treated with Versajet^®^ and Biobrane^®^ or Acticoat^®^	– Patients referred but not admitted – Patients receiving treatment other than Versajet^®^ and Biobrane^®^ or Acticoat^®^

Data were analyzed using IBM SPSS Statistics for Windows version 26 (IBM Corp, New York USA) and are presented as mean ± standard deviation. A two-tailed Student's *t*-test was performed for the continuous variables; for categorical response variables a Fisher's exact test was used. The study was supported by Buckinghamshire NHS Trust as a service evaluation study. Research ethics was not required as previous treatments that followed trust protocol were being evaluated.

## Results

The dataset included all children admitted to the ward with a burn injury between October 2014 and September 2017. All 107 children admitted met our inclusion criteria. There were 37 patients (34.6%) treated surgically with Versajet^®^ debridement and Biobrane^®^ dressings; and 70 (65.4%) patients treated conservatively with Mepitel^®^ and Acticoat^®^. Most of the pediatric patients in our study were pre-school (Versajet^®^ and Biobrane^®^ vs. Mepitel^®^ and Acticoat^®^: mean age 3.25[±0.93] vs. 2.79[±0.85]). The overwhelming majority (*n* = 105 98.1%) had scalds whilst one patient had flame burns and another chemical burns (Table 2).

**Table 2:** Patient demographics.

Variable	No. (%)/mean ± standard deviation	
	Versajet^®^ + Biobrane^®^ (*n* = 13)	Non-adherent dressing + Acticoat^®^ (*n* = 21)
Age (year)	3.25 [±0.93]	2.79 [±0.85]
Gender		
Male	18 (48.6%)	46 (65.7%)
Female	19 (51.4%)	24 (34.3%)
Total Body Surface Area %	6% [±0.77]	7% [±1.32]
Mechanism of burn		
Scald	36 (97.3%)	69 (98.6%)
Chemical	0	1 (1.4%)
Flame	1 (2.7%)	0
Further treatments	16 (43.2%)	10 (14.3%)

**Figure 1:**
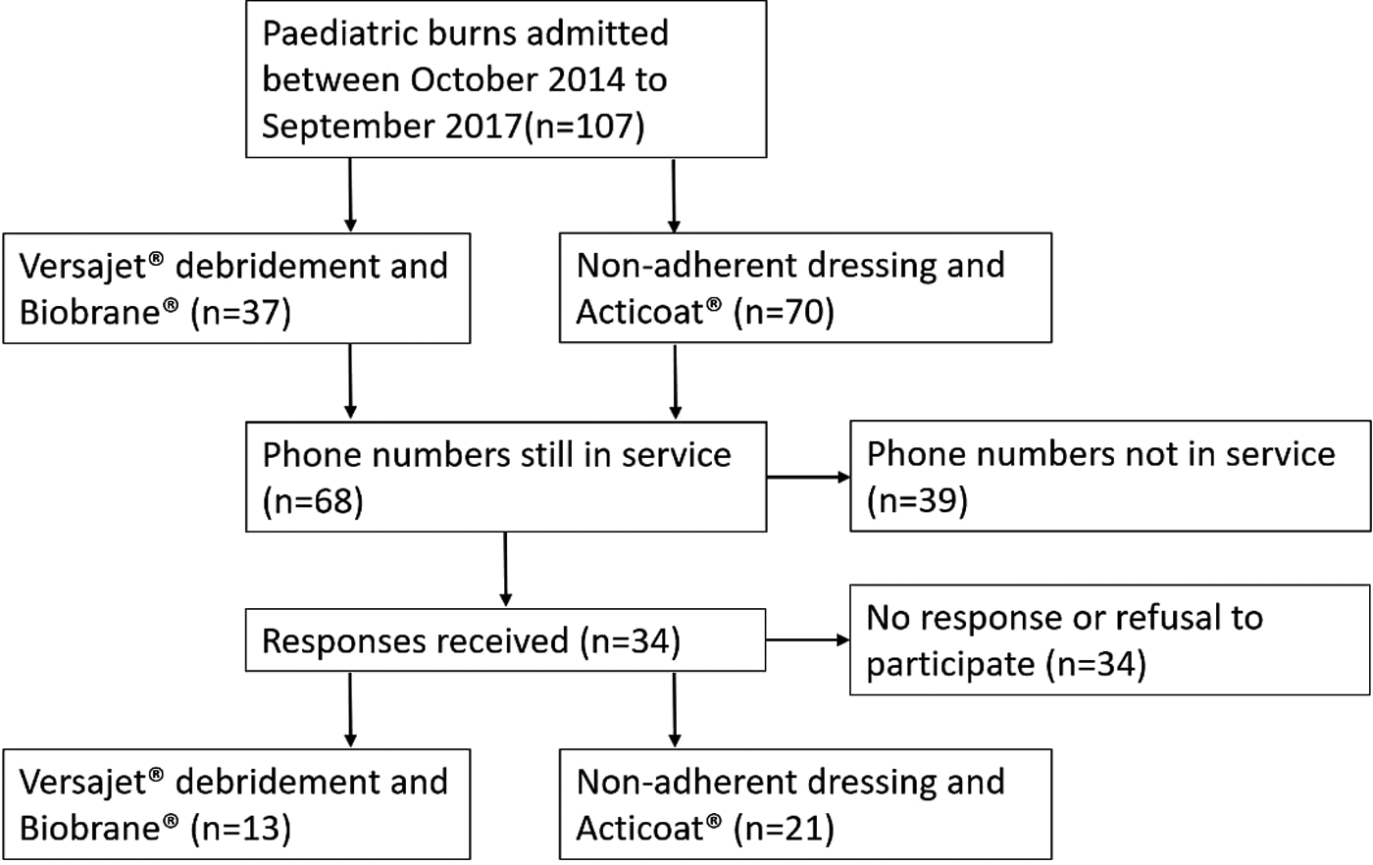
Flowchart of patient responses.

Telephone interviews resulted in a total of 34 responses (Versajet^®^ and Biobrane^®^ vs. Acticoat^®^: 13 vs. 21); 39 of the phone numbers contacted were no longer in service and a further 34 phone numbers resulted in no response or declined to participate (Figure 1).

For the responses received by telephone interview, the average interval post-injury was three years (Versajet^®^ and Biobrane^®^ vs. Acticoat^®^: 34.19[±7.71 months] vs. 36.85[±7.36 months]). As all patients were 8 years old or younger, the BBSIP_0–8_ was used (see Appendix).

Of the 58 questions that make up the BBSIP_0–8_, a two-tailed students *t*-test was performed on the results from 57 of them (Table 3). For 31 of the 57 questions, both treatment groups scored the same lowest possible score indicating no impact on the child's life/best possible outcome. For the remaining 26 questions, there was no statistically significant difference observed between the groups. The lower and upper 95% confidence intervals spanned across zero in all questions.

**Table 3:** Long term BBSIP outcomes for patients.

Variable	No.		95% Confidence Intervals		
	Versajet^®^ + Biobrane^®^ (*n* = 37)	Non-adherent dressing + Acticoat^®^ (*n* = 70)	*p* value	Lower	Upper
Overall, how much do your child's burn scars impact on their life now?^a^	1.23 [±0.60]	1.00 [±0]	0.84	−0.03297	0.49451
Itch, pain, sensitivity to touch, or other sensations from your child's scars^a^	1.31 [±1.11]	1.05± [0.22]	0.301	−0.24379	0.76393
Physical scar symptoms (like thick, tight scars)^a^	1.08 [±0.28]	1.38 [±0.80]	0.2	−0.77738	0.16932
Scar treatments (like pressure garments, exercises, creams)^a^	1.46 [±1.20]	1.05 [±0.22]	0.13	−0.12795	0.95579
School, play and daily activities^a^	1.00 [±0]	1.00 [±0]	0	N/A	N/A
Peer relationships and social interaction^a^	1.00 [±0]	1.00 [±0]	0	N/A	N/A
Your child's emotional reactions or mood^a^	1.15± [0.55]	1.00 [±0]	0.209	−0.09033	0.39803
Your child's appearance^a^	1.00 [±0]	1.14 [±0.48]	0.292	−0.41456	0.128841
During the last week, how often has your child reported itch, pain or other sensations or shown signs of sensations in their scars (like scratching, grabbing at their scars or facial grimaces)?^a^	1.31 [±1.11]	1.05 [±0.22]	0.301	−0.24379	0.76393
During the last week, on average how many times each day did your child scratch or rub their scars more than their normal skin?^a^	1.15 [±0.55]	1.00 [±0]	0.209	−0.9033	0.39803
During the last week, how many times did your child scratch or rub their scars so much that other problems happened to their scar (like wounds opened or sores developed)?^a^	1.00 [±0]	1.00 [±0]	0	N/A	N/A
Rate the severity of sensitivity of your child's burn scars to be to light touch or clothing.^a^	0.54 [±1.40]	0.19 [±0.51]	0.304	−0.33004	1.02601
Moving easily^a^	1.00 [±0]	1.00 [±0]	0	N/A	N/A
Climbing during play or up or down stairs^a^	1.00 [±0]	1.00 [±0]	0	N/A	N/A
Walking short distances^a^	1.00 [±0]	1.00 [±0]	0	N/A	N/A
Getting in and out of a chair^a^	1.00 [±0]	1.00 [±0]	0	N/A	N/A
Physical activities like swimming, riding a bike, ball games or sport^a^	1.00 [±0]	1.00 [±0]	0	N/A	N/A
Schoolwork^a^	1.00 [±0]	1.00 [±0]	0	N/A	N/A
Play^a^	1.00 [±0]	1.00 [±0]	0	N/A	N/A
Dressing and undressing^a^	1.00 [±0]	1.00 [±0]	0	N/A	N/A
Showering or bathing^a^	1.00 [±0]	1.00 [±0]	0	N/A	N/A
Eating or drinking^a^	1.00 [±0]	1.00 [±0]	0	N/A	N/A
Self-care activities (like brushing their teeth, doing their hair)^a^	1.00 [±0]	1.00 [±0]	0	N/A	N/A
Getting to sleep^a^	1.00 [±0]	1.00 [±0]	0	N/A	N/A
Staying asleep^a^	1.00 [±0]	1.05 [±0.22]	0.44	−0.17163	0.07639
Your childs daily routine^a^	1.00 [±0]	1.00 [±0]	0	N/A	N/A
Developing new skills or becoming more independent^a^	1.00 [±0]	1.00 [±0]	0	N/A	N/A
Your child's friendships or interaction with children their age^a^	1.00 [±0]	1.00 [±0]	0	N/A	N/A
Your child's interaction with family members^a^	1.00 [±0]	1.00 [±0]	0	N/A	N/A
Family activities (such as meals or outings)^a^	1.00 [±0]	1.05 [±0.22]	0.44	−0.17163	0.07639
Parent bothered by appearance of child's scars^a^	1.08 [±0.28]	1.14 [±0.48]	0.655	−0.3638	0.23193
Parent bothered by the look of your child's worst scar^a^	1.23 [±0.44]	1.10 [±0.30]	0.292	−0.12232	0.39338
Parent bothered by looks or comments you or your child got from other people because of your child's scars^a^	1.00 [±0]	1.05 [±0.22]	0.44	−0.17163	0.07639
How bothered has your child been by the appearance of their scars, during the last week^a^	1.00 [±0]	1.00 [±0]	0	N/A	N/A
Irritable or cranky^a^	1.15 [±0.55]	1.00 [±0]	0.209	−0.09033	0.39803
Anxious or nervous^a^	1.00 [±0]	1.00 [±0]	0	N/A	N/A
Worried^a^	1.00 [±0]	1.00 [±0]	0	N/A	N/A
Sad^a^	1.00 [±0]	1.00 [±0]	0	N/A	N/A
Angry^a^	1.00 [±0]	1.00 [±0]	0	N/A	N/A
Embarassed or self-conscious^a^	1.00 [±0]	1.05 [±0.22]	0.44	−0.17163	0.07639
Upset^a^	1.00 [±0]	1.00 [±0]	0	N/A	N/A
Worst part – Tight^a^	1.00 [±0]	1.00 [±0]	0	N/A	N/A
Worst part – Thick^a^	1.69 [±1.25]	1.33 [±0.66]	0.28	−0.30664	1.02458
Worst part – Wrinkled^a^	1.31 [±0.63]	1.10 [±0.30]	0.194	−0.11348	0.53839
Worst part – Dry^a^	1.15 [±0.55]	1.10 [±0.44]	0.734	−0.28944	0.40666
Worst part – Hard^a^	1.15 [±0.55]	1.00 [±0]	0.209	−0.09033	0.39803
Worst part – Rough^a^	1.00 [±0]	1.00 [±0]	0	N/A	N/A
Worst part - A different colour^a^	2.08 [±0.95]	1.71 [±0.72]	0.216	−0.22258	0.94786
Did your child have open wounds or sores in their scars, during the last week?^a^	0	0	0	N/A	N/A
Parent worry - whether the lack of your child's scars will bother them in the future?^a^	1.23 [±0.44]	1.10 [±0.30]	0.292	−0.12232	0.39338
Parent worry - the effect of your child's scars on other family members^a^	1.08 [±0.28]	1.00 [±0]	0.209	−0.4517	0.19901
Parent worry - the way others treated your child^a^	1.00 [±0]	1.10 [±0.30]	0.265	−0.26618	0.0757
Parent - ability to work, study, or complete household jobs^a^	1.00 [±0]	1.00 [±0]	0	N/A	N/A
Parent - relationship with family members^a^	1.00 [±0]	1.00 [±0]	0	N/A	N/A
Parent - you getting together with friends^a^	1.00 [±0]	1.00 [±0]	0	N/A	N/A
Parent – mood^a^	1.00 [±0]	1.05 [±0.22]	0.44	−0.17163	0.7639
Parent - family routine^a^	1.00 [±0]	1.05 [±0.22]	0.44	−0.17163	0.7639

^a^ Data presented as mean ± standard deviation.

Part 7 of the BBSIP focuses on physical symptoms with question 15 asking caregivers to rate how much the child's worst area of scar was affected by a physical property in the last week.

**Figure 2:**
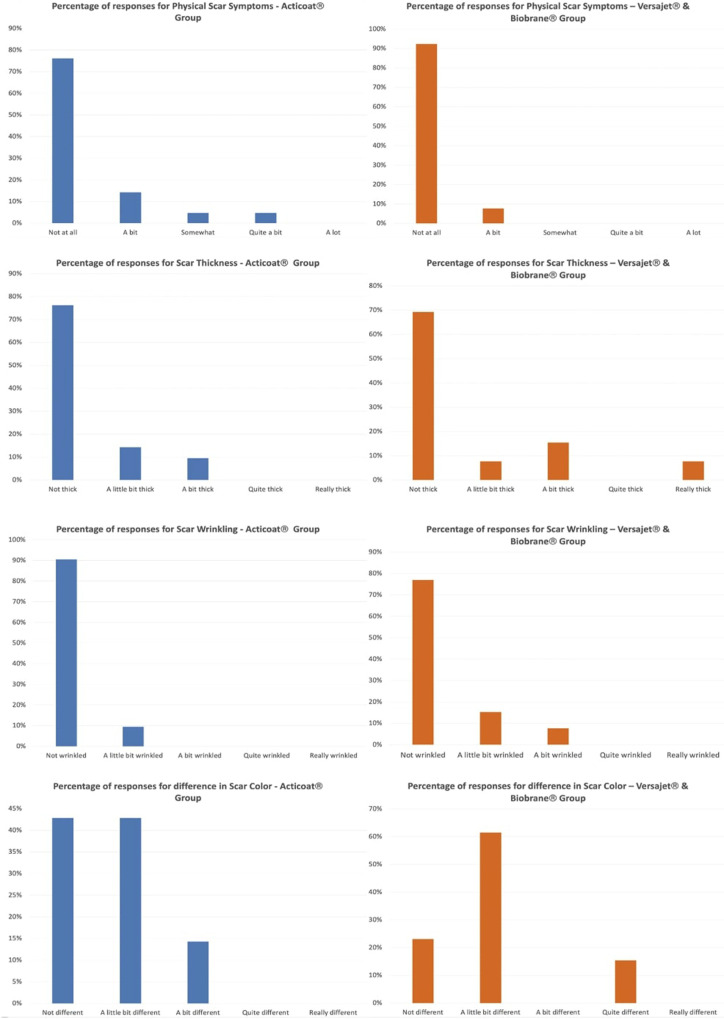
Percentage responses for worst scar physical properties.

Physical properties of thickness (question 15B), wrinkling (question 15C) and difference in color (question 15G) demonstrated the larger differences (Figure 2). Responses for question 15B relating to scar thickness in the conservative treatment group were: 16 (76%) conservative vs. 9 (69%) surgical reported not thick, 3 (14%) conservative vs. 1 (8%) surgical reported a little bit thick, 2 (10%) reported a bit thick in conservative and 1 (8%) reported really thick in surgical. Question 15C focused on wrinkling with responses as; 19 (91%) conservative, vs. 10 (77%) surgical reported not wrinkled, 2 (9%) conservative vs. 2 (15%) reported a little bit wrinkled with 1 (8%) report of a bit wrinkled in the surgical group. Scar colour asked in question 15G had the largest difference in responses; 9 (43%) conservative vs. 3 (23%) surgical reported not different, 9 (43%) conservative vs. 8 (62%) surgical reported a little bit different, 3 (14%) conservative reported a bit different and 2 (15%) surgical reported quite different. In the surgical treatment group, 6 of the 13 had further treatments in clinic, whilst 3 of the 21 children in the conservative group had further treatments. Question 14 in part 7 of the BSIP_0–8_ asked to describe the worst part of the child's scar by anatomical location. The Fishers exact test was used for any correlation between worst areas of burn and treatment group. No statistically significant difference was demonstrated between the two groups (Fishers Exact Test = 0.817).

## Discussion

This study did not identify any statistically significant differences in longer-term scar outcomes as assessed by the BBSIP_0–8_ in pediatric burn patients treated with surgical Versajet^®^ debridement and Biobrane^®^ vs. those treated conservatively with Acticoat^®^ and non-adherent dressings. For over half of the questions asked as part of the BBSIP, all responses in both groups were the lowest possible score indicating the least/no impact of the scar on the child's life. For part 3 of the BBSIP_0–8_ which focuses on school, play and daily activities, 14 of the 15 questions in this section all scored the lowest possible score in both groups. Part 6 of the BBSIP_0–8_ focuses on emotional reactions; we found that 5 out of the 7 questions in the sections had scored the lowest possible score in both groups (irritable or cranky and self-conscious). The area that had the most variation was part 7 of the BBSIP_0–8_ which focuses on physical symptoms. Questions focused on the worst areas of scar tightness and roughness scored the lowest possible in both groups. Thickness, wrinkling, dryness, hardness and difference in color all had the greater variety of responses in both groups. These properties are assessed as part of the POSAS patient scale referred to as scar color, stiffness, thickness and irregularity. From our data, we can conclude that between both treatment groups there is no significant long-term difference in the scarring outcome; with most variation observed in differences in scar colour, thickness, hardness, stiffness and irregularity compared to normal skin.

Burns specialists will use clinical judgement to determine whether or not to debride a burn, weighing up the risks of treating surgically vs. conservatively. There can be some flexibility to choose whichever approach will get the child healed fastest, acknowledging that the longer-term scarring outcomes would likely be the same. One of the caveats of the study is that potentially the children treated through different methodologies had different depths, although this is something that we aimed to control for. Our results revealed that a higher proportion of the children treated surgically underwent further treatments in scar clinic compared to those treated conservatively. This could be due to deeper, more severe burns in those treated surgically. Despite this, longer term scarring outcomes were similar.

There are multiple subjective outcome measures to assess the results of a scar. The most commonly used in academic literature include the VSS and its various modifications, the MSS and the POSAS; however, none of these are specifically targeted at children. The POSAS is a unique outcome assessment tool as it includes both an assessor score and a patient reported score and has been used in pediatric longer-term burn scar studies by conducting telephone interviews with the parents of children (9). The POSAS patient section assesses the scars on pain, itch, color, stiffness, thickness and irregularity. The defined criteria for a PROM's quality is based on content validity, internal consistency, criterion validity, construct validity, reproducibility, longitudinal validity, responsiveness, floor and ceiling effects and interpretability (29). The defined COSMIN criteria can be used to assess the methodological quality of studies assessing a PROM (30). A suitable PROM for children with burn scars would include assessment of how the scar affects all elements related to quality of life (physical, mental and social well-being) (16). Pediatric specific PROMs would ideally use language that is age-appropriate, not include elements irrelevant to children (such as driving and financial) and be focused on factors important in a child's development such as play. For younger children with a burn injury, the PROM would be more appropriate to be aimed at the primary caregiver of the child. Given the aesthetic as well as functional impact of scarring, a suitable PROM would be able to distinguish from patients that have undergone surgery and those that haven't (31, 32). It would additionally allow for comparison of techniques and identify patients likely to benefit from the procedure (32).

This study is the first to utilize the BBSIP_0–8_ as a longer-term scar outcome tool. Tyack et al 2019 described the value of the BBSIP_0–8_ in the acute post burn phase and that more studies would be required to establish the BBSIP_0–8_ role for longer term scar outcomes (15). The small sample size means the study may not be adequately powered and is potentially at risk of type II error. The clinical entries used as part of the data collection would not always include the depths of the burn injury and language used to describe depth was not uniform. Additionally, there is a degree of subjectivity when assessing depths of burns clinically. Telephone interviews have generally been regarded as less-attractive than face-to-face interviews and physical questionnaires (33). Negatives of telephone interviews in research have been reported as a lack of visual cues can result in a loss of contextual and non-verbal data which in turn may lead to lower quality data (33). The BBSIP_0–8_ is a physical questionnaire designed to be filled in by the caregiver of the patient. Whether or not telephone interviews result in lower quality data compared to physical interviews or questionnaires remains a matter of debate (33).

In conclusion, no difference was found in the long term scar outcomes as assessed by the BBSIP_0–8_ in those treated surgically with Versajet^®^ debridement and Biobrane^®^ compared to those treated conservatively with Acticoat^®^ and non-adherent dressings. The BBSIP and its various versions are the only full PROM for burns scars widely available. It has value as a longer-term scar assessment tool, but a shorter more focused version may be of more value in clinic.

## Contribution to the field statement

Burn injuries in children are a common occurrence accounting for approximately ¼ of all burn presentations. The general initial aim of burn management is to resuscitate if necessary and reduce the TBSA/depth of the burn by cooling the area down. Following this, a decision based upon clinical assessment is made on whether the burn can be treated conservatively with dressings or whether surgical intervention is required. The decision to operate is based upon the depth of the burn and what will likely cause the burn to heal fastest. As children are often discharged after a six week follow up the long-term appearance of the scar is relatively unknown unless they re-present with a problematic scar. By using the world's only available patient reported outcomes measure specifically aimed at assessing pediatric burn scars; we have demonstrated there is no statistically significant difference in the long-term scarring outcomes in pediatric burns treated conservatively with Mepitel^®^ and Acticoat^®^ vs. those treated surgically with Versajet^®^ debridement and Biobrane^®^. The Brisbane Burn Scar Impact Profile aimed at children has value in assessing long-term scars, but a limitation is the length of the questionnaire. We propose that a shorter more focused version may be more valid in a time pressured clinical environment.

## Data Availability

The original contributions presented in the study are included in the article/Supplementary Material, further inquiries can be directed to the corresponding author/s.
